# A novel method for assessing the renal biopsy specimens using an activatable fluorescent probe

**DOI:** 10.1038/s41598-020-69077-w

**Published:** 2020-07-21

**Authors:** Takuji Iyama, Tomoaki Takata, Kentaro Yamada, Yukari Mae, Sosuke Taniguchi, Ayami Ida, Masaya Ogawa, Marie Yamamoto, Shintaro Hamada, Satoko Fukuda, Tsutomu Kanda, Takaaki Sugihara, Hajime Isomoto, Yasuteru Urano

**Affiliations:** 1 Division of Gastroenterology and Nephrology, Tottori University Faculty of Medicine, 36-1, Nishimachi, Yonago, Tottori 683-8504 Japan; 2 Laboratory of Chemical Biology and Molecular Imaging, Graduate School of Medicine, The University of Tokyo, 7-3-1 Hongo, Bunkyo-ku, Tokyo, 113-0033 Japan

**Keywords:** Medical research, Nephrology, Urology

## Abstract

Gamma-glutamyl hydroxymethyl rhodamine green (gGlu-HMRG) is an activatable fluorescent probe that can be activated by γ-glutamyltranspeptidase (GGT). The expression of GGT in the kidney, which is one of the major organs exhibiting enhanced GGT expression, is exclusively localised to the cortex. Here, we aimed to investigate the feasibility of gGlu-HMRG as a probe for the on-site assessment of renal biopsy specimens. gGlu-HMRG fluorescent probe was applied to the renal proximal tubular epithelial cells and cortical collecting duct cells in vitro, mouse kidneys ex vivo, and human biopsy specimens. In addition, the fluorescence intensities in the cortex and the medulla were comparatively evaluated in the biopsy specimens. The fluorescence signal was rapidly detected in the renal proximal tubular epithelial cells, whereas that in the cortical collecting duct cells was not detected. The fluorescence signal was detected in the mouse kidneys ex vivo without markedly affecting the tissue morphology. In the human biopsy specimens, the fluorescence signal in the cortex was significantly distinct from that in the medulla (p < 0.05). Thus, this fluorescent probe can be used to distinctly identify the renal cortex in the biopsy specimens.

## Introduction

Renal biopsy is one of the most important procedures in clinical nephrology. In most cases, a percutaneous ultrasound-guided renal biopsy is performed. The histological analysis of biopsy tissues is important for the diagnosis and prognosis assessment of renal diseases, as well as determining the treatment strategy^1^. Although renal biopsy is an undisputed diagnostic procedure, nephrologists/urologists must consider both the benefits and potential complications of renal biopsy. The complications associated with percutaneous renal biopsy include haemorrhage, blood transfusion, and loss of real function. A previous meta-analysis has reported that renal biopsy is associated with a considerable rate of bleeding complications^2^. Thus, the number of punctures should be minimised and it is important to evaluate the quality of the obtained tissues to avoid unnecessary puncture.

Gamma-glutamyl hydroxymethyl rhodamine green (gGlu-HMRG) is a recently developed activatable fluorescent probe^3^. gGlu-HMRG is non-fluorescent but emits fluorescence upon activation by γ-glutamyltranspeptidase (GGT)-mediated enzymatic catalysis. As some of the cancer cells exhibit enhanced expression of GGT, gGlu-HMRG was originally developed to detect cancers. Previously, gGlu-HMRG was used to detect resected colon cancer in a mouse model^4^. Additionally, gGlu-HMRG could detect pancreatic cancers in biopsy specimens obtained from endoscopic ultrasound-guided fine-needle aspiration^5^. Furthermore, gGlu-HMRG could diagnose *Helicobacter pylori* infection in the gastric biopsy specimen^6^. The novel gGlu-HMRG-based imaging technique can be applied to evaluate various biopsy specimens other than detecting cancer. The advantage of gGlu-HMRG is that this probe emits fluorescence immediately after activation. Therefore, it would be a powerful tool for rapid evaluation of the renal biopsy specimen leading to the acquisition of sufficient number of glomeruli for the diagnosis of glomerulopathy.

GGT is an enzyme expressed in the plasma membrane of various organs. In clinical practice, GGT is commonly used as a marker for liver disease or obstruction of biliary tract^7^. GGT is abundantly expressed in the kidney with the cortex exhibiting higher GGT activity than the medulla or papilla^8,9^. It is important to obtain renal biopsy tissues containing adequate glomeruli for histological assessment. As glomeruli are localised exclusively in the renal cortex, gGlu-HMRG may aid in determining the glomerulus content in the biopsy specimen. Rapid evaluation of the biopsy specimen would result in reducing unnecessary puncture and accurate diagnosis. In this study, we investigated the feasibility of this activatable fluorescent probe for on-site evaluation of renal biopsy specimens.

## Results

### Fluorescence imaging of cell culture

**Figure 1:**
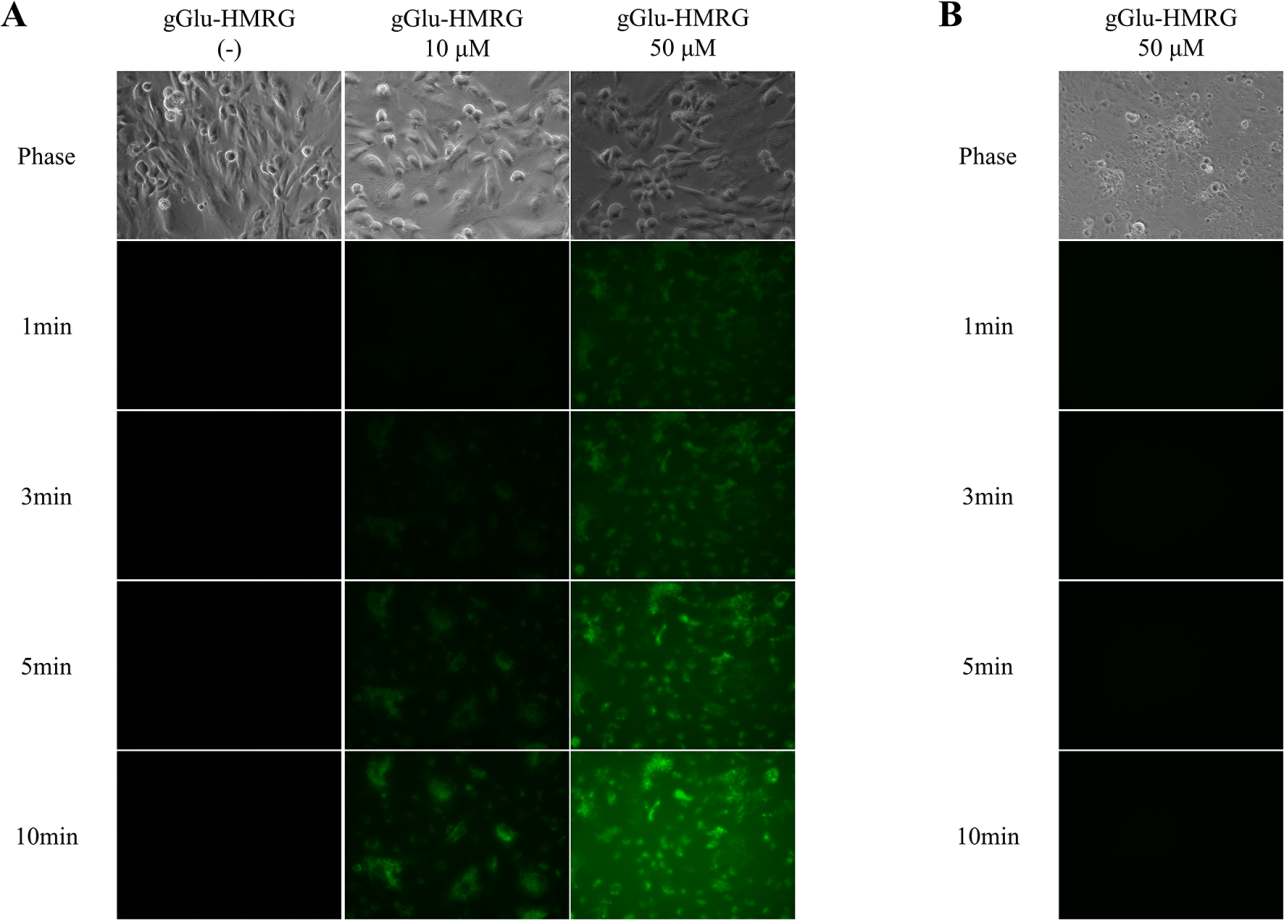
In vitro experiments with RPTECs and M-1 cells. Phase-contrast and fluorescence images of (**A**) RPTECs and (**B**) M-1 cells. gGlu-HMRG was diluted in phosphate-buffered saline and applied at concentrations of 10 or 50 μM. Fluorescence images were captured at 1, 3, 5, and 10 min post-gGlu-HMRG administration. The fluorescence signal was detected only in the RPTECs. gGlu-HMRG; γ-glutamyl hydroxymethyl rhodamine green, RPTEC; renal proximal tubule epithelial cell, M-1; mouse cortical collecting duct.

GGT is highly expressed in the proximal tubule, which is located in the renal cortex^8^. Thus, we first investigated the fluorescence signal in the renal proximal tubule epithelial cells (RPTECs) after treatment with gGlu-HMRG. We also investigated the fluorescence in cortical collecting duct cells as the negative control. The RPTECs and collecting duct cells were treated with 10 or 50 μM gGlu-HMRG. The fluorescence images were obtained at 1, 3, 5, and 10 min post-gGlu-HMRG administration. The fluorescence signal was clearly detected in the RPTECs, whereas it could not be detected in the cortical collecting duct cells (Fig. 1A,B). The fluorescence signal in the RPTECs was stronger when gGlu-HMRG was applied at higher concentration and the intensity gradually increased immediately after the administration of gGlu-HMRG (Fig. 1).

### Fluorescence imaging of mouse kidneys

**Figure 2:**
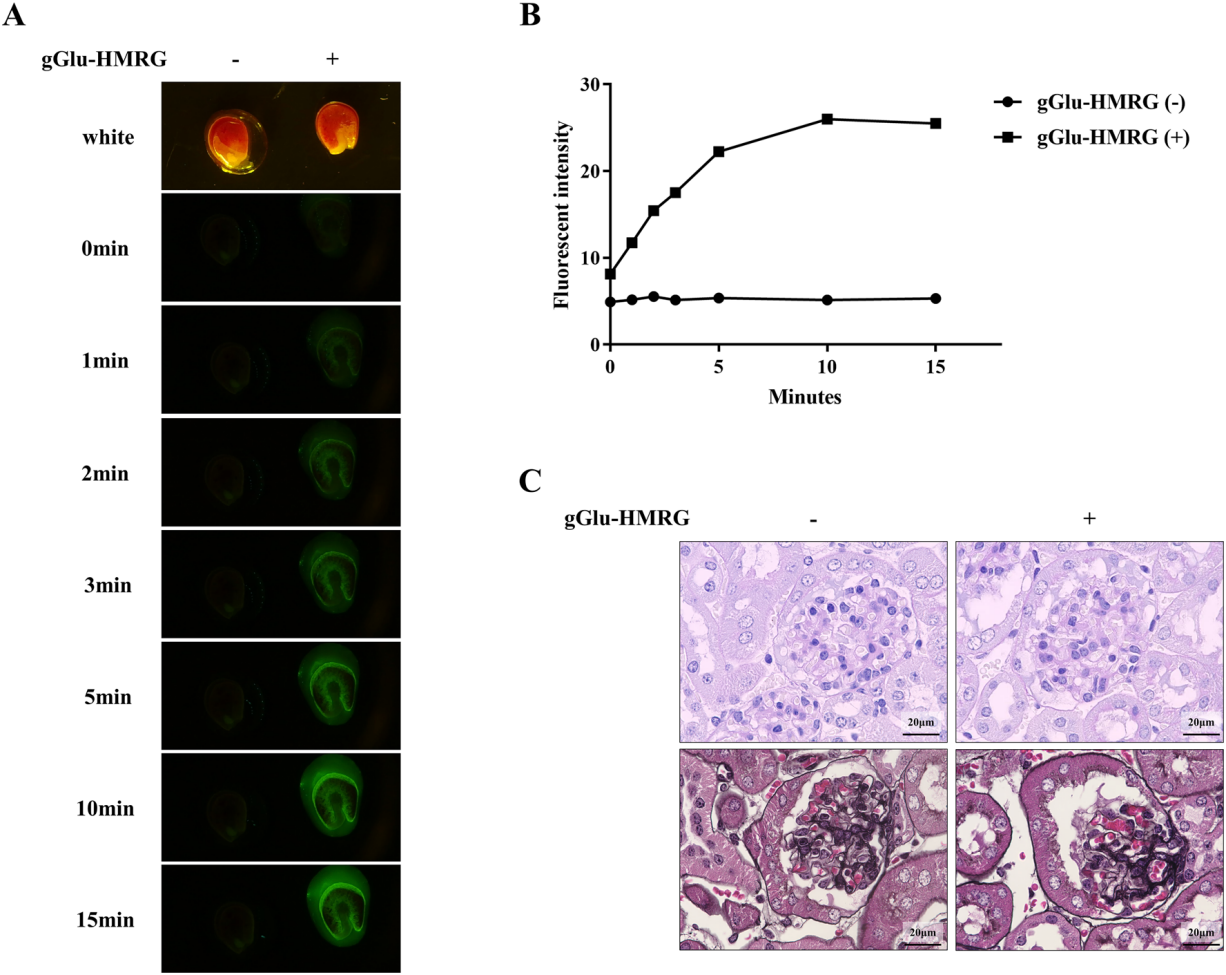
Ex vivo analysis of mouse kidneys. (**A**) Fluorescence images of the mouse kidney sections incubated with gGlu-HMRG. (**B**) The time-course of changes in the fluorescence intensities. The intensity gradually increased and plateaued at 10 min post-gGlu-HMRG administration. (**C**) Representative images of PAS and PAM staining obtained from paraffin-embedded mouse kidney sections incubated with gGlu-HMRG or PBS. There was no marked change in the two groups. gGlu-HMRG; γ-glutamyl hydroxymethyl rhodamine green, PAS; periodic acid-Schiff, PAM; periodic acid-methenamine silver, PBS; phosphate-buffered saline.

Next, the mouse kidneys were treated with gGlu-HMRG ex vivo. The kidneys from C57BL6/J mice were transversely sectioned. The sectioned kidneys were rinsed with phosphate-buffered saline (PBS) at pH 7.4 and treated with 50 μM gGlu-HMRG. The fluorescence signal was detected at 1 min post-gGlu-HMRG administration. The fluorescence intensities gradually increased and plateaued at 10 min (Fig. 2A,B). Further, the effect of gGlu-HMRG treatment on the morphology of the tissue was evaluated by immunohistochemistry. There was no marked change in the morphology of gGlu-HMRG-treated and negative control tissues. This suggested that gGlu-HMRG had little influence on the histological assessment of renal disease (Fig. 2C). In addition, we have confirmed that gGlu-HMRG had no relevant effect on immunofluorescent staining or electron microscopy (Supplementary Fig. S1 online).

### Fluorescence imaging of renal biopsy specimens

**Figure 3:**
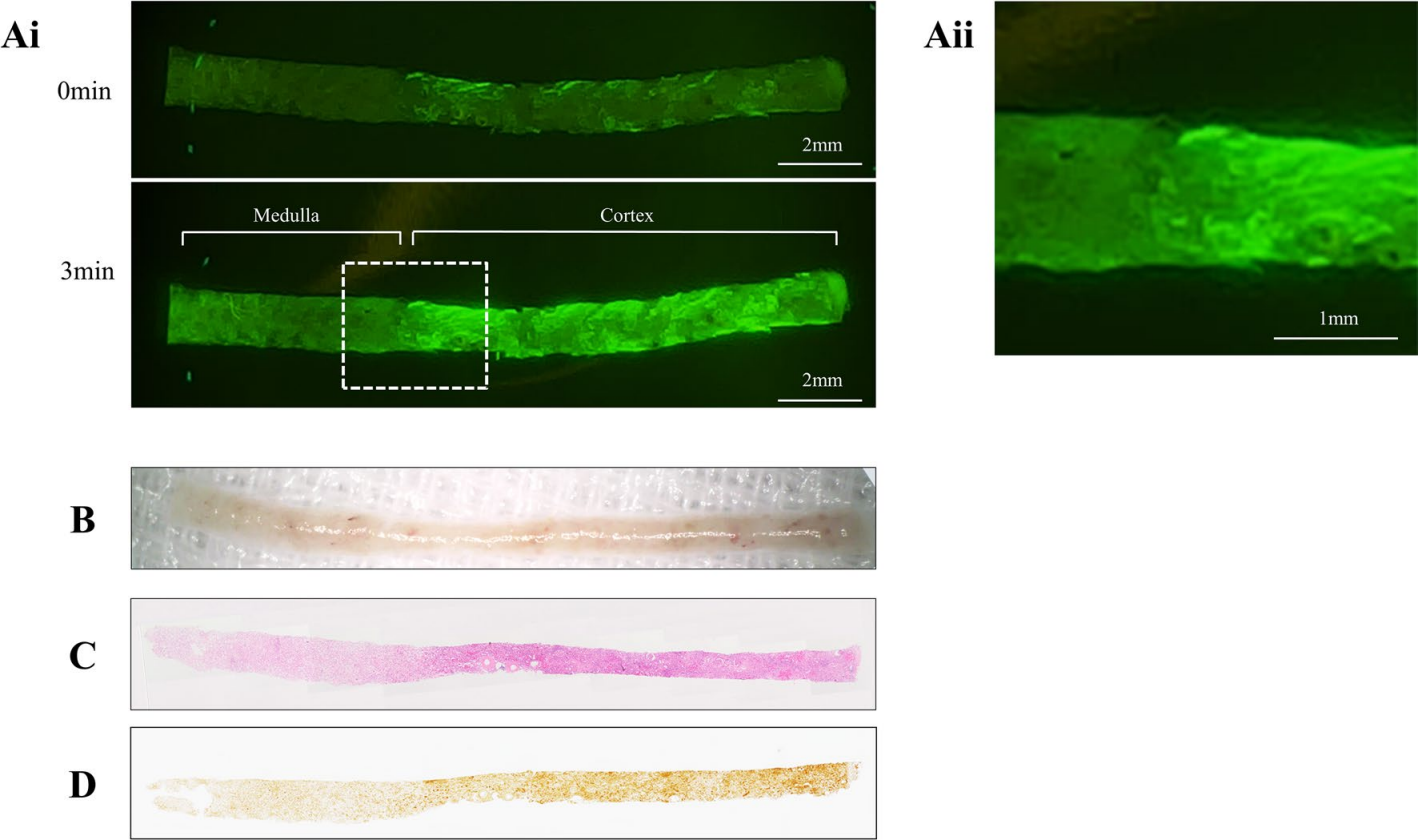
Representative fluorescence images of the human biopsy specimen. (**Ai**) Fluorescence images captured at 0 and 3 min post-50 μM gGlu-HMRG administration. (**Aii**) Magnified fluorescence image of the cortico-medullary junction. (**B**) The image captured from the same specimen using a magnifying lens under white light. Cortico-medullary junction, which was clearly detected in the fluoserscence images, is obscure under magnified lens. (**C**) PAS staining image of the same specimen. (**D**) The same specimen was subjected to GGT immunostaining. The region with intense fluorescence intensity corresponds to the cortex and the area with a strong GGT signal. gGlu-HMRG; γ-glutamyl hydroxymethyl rhodamine green, PAS; periodic acid-Schiff, GGT; γ-glutamyltranspeptidase.

**Figure 4:**
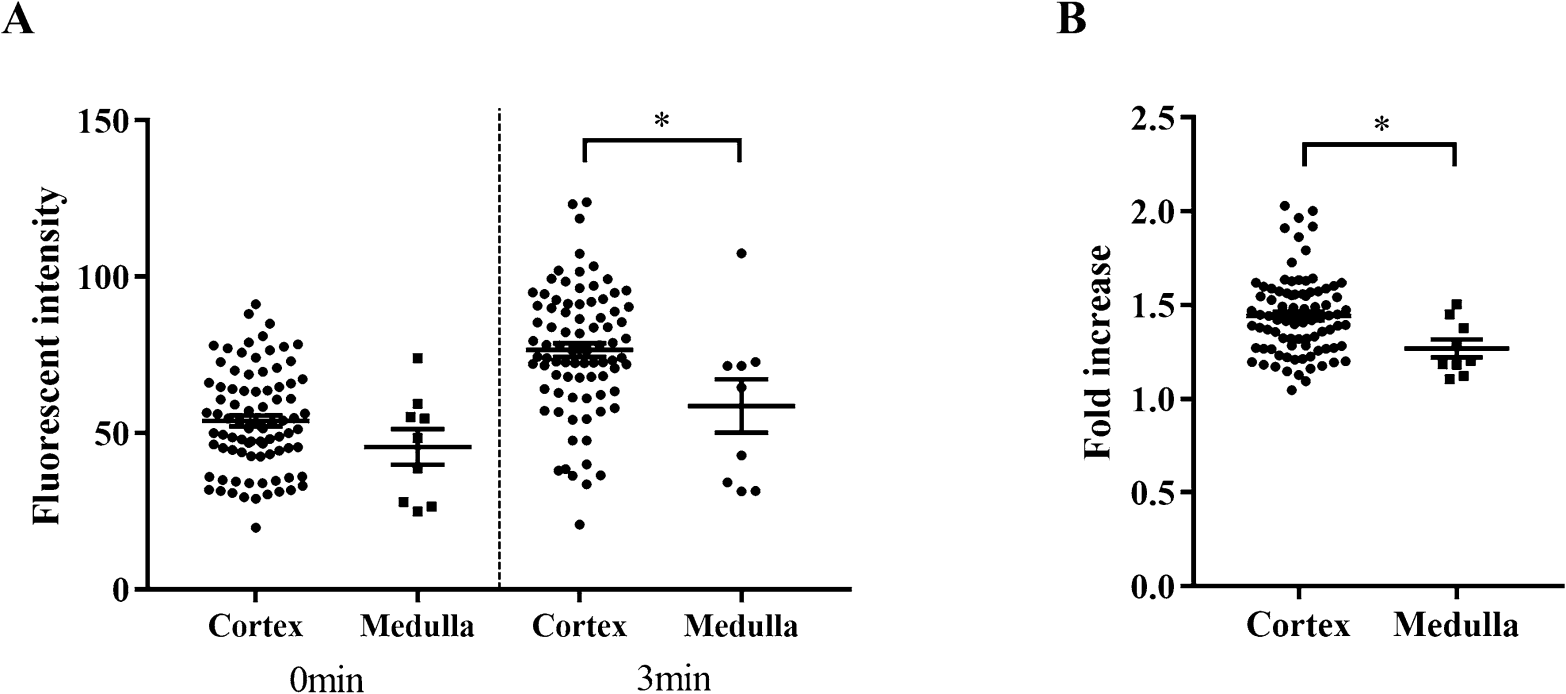
Fluorescence intensities in the cortex and medulla. (**A**) Fluorescence intensities in each 8 parts from 12 patients at 0 and 3 min post-50 μM gGlu-HMRG administration. Each part was divided into the cortex or medulla according to the periodic acid-Schiff staining. Data were analysed by two-way analysis of variance with post hoc Sidak’s test. (**B**) Fold increase in the intensities after 3 min incubation with gGlu-HMRG. Data were analysed by unpaired t test. Bars indicate average ± standard error of mean (SEM). *P < 0.05. gGlu-HMRG; γ-glutamyl hydroxymethyl rhodamine green.

The fluorescence images of renal biopsy specimens were obtained from 12 patients (Table 1). The fluorescence signal was detected in all examined specimens. The representative fluorescence images are shown in Fig. 3. The fluorescence signal was detected immediately after administration and the intensity increased at 3 min post-gGlu-HMRG administration. Additionally, the fluorescence intensity varied segmentally within the specimen, which was suggestive of the cortico-medullary junction (Fig. 3A). However, the cortico-medullary junction was not clearly visible under magnified lens (Fig. 3B). The histological examination using periodic acid-Schiff (PAS) staining revealed that the cortex exhibited strong fluorescence intensity, whereas the medulla exhibited weak fluorescence intensity (Fig. 3A,C). These findings were further confirmed by immunohistochemical staining for GGT. The area exhibiting high fluorescence intensity, which is the cortex region, also exhibited high GGT expression (Fig. 3D). In contrast to the fluorescence image, the cortico-medullary junction was obscure under the magnified lens. Fluorescence microscopy analysis also revealed that the glomerulus was devoid of fluorescence (Supplementary Fig. S2 online). Further, the fluorescence intensity in the kidneys of 12 patients was quantified to investigate the feasibility of gGlu-HMRG as a probe to distinguish the cortex and medulla. There was no significant difference in the fluorescence intensity of cortex and medulla at 0 min. However, the cortex exhibited significantly enhanced fluorescence intensity at 3 min (p < 0.05). The fold increase in the fluorescence intensity in the cortex was higher than that in the medulla (Fig. 4). These results indicated that the renal cortex and medulla could be distinguished by assessing the fluorescence intensity. In fact, glomeruli were obviously distributed in the cortical area, where stronger fluorescence was observed (Supplementary Fig. S3 online).

**Table 1:** Patient characteristics and pathological diagnosis of renal biopsy.

Characteristic	Value
N	12
Age (years)	64.5 (32–85)
Sex (male/female)	10/2
**Pathological diagnosis of renal biopsy**	
Sclerosing glomerulosclerosis	3 (25.0)
Mesangial proliferative glomerulonephritis	2 (16.7)
Crescentic glomerulonephritis	2 (16.7)
Minor glomerular abnormality	2 (16.7)
Amyloidosis	1 (8.3)
Diabetic nephropathy	1 (8.3)
Interstitial nephritis	1 (8.3)

Data are presented as the median (range) or n (%).

## Discussion

In this study, we demonstrated that gGlu-HMRG application generated fluorescence signals in the renal proximal tubular cells in vitro, mouse kidneys ex vivo, and human renal biopsy specimens. The cortex and medulla could be easily distinguished by assessing the fluorescence intensity. This novel gGlu-HMRG-based imaging technique can be a help for on-site assessment of biopsy specimens and is feasible for application in clinical nephrology.

Here, in vitro studies demonstrated that the fluorescence signals are detected in the renal proximal tubular cells but not in the collecting duct cells after treatment with gGlu-HMRG. These findings concur with those of the previous study that investigated the GGT activity in each tubular segment. The renal proximal tubule exhibited the highest GGT activity when compared to the other segments of the nephron, including glomerulus, thick ascending limb of Henle’s loop, distal convoluted tubule, and collecting duct^8^. Previous in vitro studies have demonstrated the feasibility of gGlu-HMRG as a probe to detect the cancer cells derived from intra-abdominal organs^3–5,10^. This technique has not been applied to normal kidneys or renal neoplasms. We provided a possible application of this fluorescence imaging to normal kidneys. Although we observed fluorescence variability in different nephron segments, application to renal neoplasms may require further investigation.

The fluorescence intensity was dependent on the gGlu-HMRG concentration and incubation duration. In vitro experiments revealed that 10 μM gGlu-HMRG required longer incubation time to detect the fluorescence signal than 50 μM gGlu-HMRG. A previous study applied 50 to 100 μM gGlu-HMRG on human biopsy specimens^5,6,10–12^ and detected the fluorescence signal within 5 min post-gGlu-HMRG administration. In this study, ex vivo analysis of the mouse kidney revealed that the fluorescence signal was clearly detected at 3 min post-gGlu-HMRG administration. The fluorescence intensity gradually increased and plateaued after 10 min. Therefore, an incubation time of 3 min and 50 μM gGlu-HMRG was sufficient for the on-site assessment of renal biopsy specimen. There were no morphological changes observed in the mouse kidneys after PAS and periodic acid-methenamine silver (PAM) staining. This indicated that the human specimens can be incubated with 50 μM gGlu-HMRG for 3 min without influencing the histological assessment. Actually, all human biopsy specimens could be pathologically diagnosed upon incubation with gGlu-HMRG. Another influencing factor may be the handling of the obtained specimens. A previous study using ovarian cancer model mice reported that pressed tumour emitted faint fluorescence when compared to the intact, rinsed, or cut tumor^11^. The fluorescence in the rinsed mouse and human specimens could be detected in the present study.

Renal biopsy is considered when glomerular diseases are suspected. In most cases, the diagnosis or treatment depends on the histological assessment of glomerulus. As glomeruli are exclusively distributed in the renal cortex^13^, it is important to obtain the biopsy specimen containing the cortex. The biopsy specimens lacking the cortex might provide insufficient information. Stereomicroscope or magnifying lens are usually used for the on-site evaluation of the specimens. However, it is often difficult to clearly identify the cortex. The novel gGlu-HMRG-based imaging technique described in this study can address this limitation. The renal proximal tubule that lies adjacent to the glomerulus is composed of the proximal convoluted tubule and proximal straight tubule, which are located mainly in the renal cortex^14^. Therefore, the fluorescence signals can be detected in the biopsy specimens containing the renal cortex after treatment with gGlu-HMRG, which was demonstrated in this study. Additionally, the renal cortex and medulla could be easily distinguished by quantifying the fluorescence intensities. Although conventional histological staining such as PAS and PAM is the standard for histological diagnosis of glomerular diseases, gGlu-HMRG can be applied to the specimens before fixation and enables to assess the quality of the specimen leading to avoid unnecessary puncture.

This study has some limitations. The pathological aetiology or renal function that may potentially influence the fluorescence signal was not evaluated in this study because of the small number of the subjects and varied aetiologies of the kidney diseases. The green fluorescence of gGlu-HMRG potentially interfere with the immunofluorescent staining for gamma globulin or immune complex, which is another important method for diagnosing glomerular diseases. However, any interference of gGlu-HMRG on immunofluorescent staining could not be observed in this study. A silicon rhodamine-based fluorescent probe, which emits red fluorescence, for detecting GGT has been developed^15^ and that would be an option for the concern about the color interference. There remains one concern that glomerulus was not stained by gGlu-HMRG, thus this fluorescent probe is not a direct indicator of the number of glomeruli contained in the specimen. We have demonstrated that the cortex, which contained a definite number of glomeruli, showed significant increase in the fluorescent intensity. In contrast, the part with weak fluorescence by gGlu-HMRG (i.e. medulla), did not contain any glomeruli.

In conclusion, we demonstrated that the activatable fluorescent probe, gGlu-HMRG, could be applied to renal biopsy specimens for distinguishing the renal cortex. Further clinical investigations are required to elucidate the fluorescence signal variations in various kidney diseases and diagnostic accuracy.

## Methods

### Activatable fluorescent probe

The activatable fluorescent probe, gGlu-HMRG, was purchased from GORYO Chemical, Sapporo, Japan. gGlu-HMRG was dissolved in dimethyl sulfoxide at 1 mM and stored at − 20 °C before use. The gGlu-HMRG stock solution was thawed on ice and diluted in PBS to the indicated concentrations and used for the analyses.

### In vitro imaging using the fluorescent probe

The RPTECs were obtained from the American Type Culture Collection (ATCC, Rockville, Maryland, USA), while the mouse cortical collecting duct (M-1) cells were purchased from KAC Co., Ltd., Tokyo, Japan. The RPTECs were maintained in Dulbecco’s modified Eagle’s medium (DMEM)/F12 supplemented with 10% foetal bovine serum, 1% l-glutamine, and 1% antibiotic G418. The M-1 cells were maintained in DMEM/F12 supplemented with 10% foetal bovine serum, 1% l-glutamine, and 5 μM dexamethasone. The cells were cultured in a humidified incubator at 37 °C in an atmosphere of 95% air and 5% carbon dioxide. Confluent cells were rinsed with PBS and incubated with gGlu-HMRG at a concentration of 10 or 50 μM. The fluorescence images were obtained using a fluorescence microscope (BZ-X710; Keyence, Osaka, Japan) with the excitation wavelength of 450–470 nm and the emission wavelength of 500–550 nm along with the phase-contrast images.

### Ex vivo imaging using the fluorescent probe

Eight-week-old C57BL6/J male mice were purchased from CLEA Japan, Inc., Tokyo, Japan for ex vivo experiments. The mice were maintained in 12-h light–dark cycle and provided with water and standard laboratory diet ad libitum. The mice were anesthetised with isoflurane and the kidneys were harvested. The kidneys were transversely sectioned in 2 mm thickness and the sections were briefly rinsed with PBS. The kidney sections were treated with 50 μM gGlu-HMRG and exposed to an excitation wavelength of 450 nm. The fluorescence images were obtained using a commercially available digital camera equipped with an optical interference filter (~ 520 nm) immediately after gGlu-HMRG administration for 15 min. The kidney sections were then fixed with 4% paraformaldehyde and embedded in paraffin. Two sections were subjected to PAS and PAM staining. The animal experiments were performed in accordance with the ethical guidelines at Tottori University. The experimental protocols were approved by the ethical committee of the animal facility at the Tottori University (approval number: h30-Y035).

### Fluorescent imaging of biopsy kidney specimens

Patients who underwent percutaneous ultrasound-guided renal biopsy were included in the study. This study was conducted in accordance with the Declaration of Helsinki and approved by the ethical committee of Tottori University hospital (approval number: 18A135). All study participants provided their informed consent. The indications of the renal biopsy were urinary abnormality or decline in renal function, which was suggestive of glomerular diseases as previously described^16^. Renal biopsy was performed using a 16-gauge biopsy gun (Acecut; TSK Laboratory, Tochigi, Japan) under ultrasound-guided puncture. The biopsy tissue was briefly rinsed with normal saline and subjected to fluorescence imaging analysis. The biopsy specimen was treated with 50 μM gGlu-HMRG and fluorescence images were obtained immediately and at 3 min post-gGlu-HMRG administration with the same setting used in the ex vivo experiments. The specimens were then fixed in formalin and embedded in paraffin. The PAS-stained section was evaluated to assess the distribution of the cortex. For GGT immunostaining, a 4-μm thick section was subjected to antigen retrieval. The section was blocked with a solution containing 3% bovine serum albumin in PBS. The section was then incubated with the rabbit anti-GGT1 antibody (1:1,000, GeneTex, Irvine, CA, USA) overnight at 4 °C. Next, the section was incubated with the donkey anti-rabbit secondary antibody (1:500, Abcam, Tokyo, Japan). The images of PAS-stained and immunostained sections were obtained using BZ-X710 microscope (Keyence).

### Fluorescence intensity analyses

For analysing the ex vivo mouse samples and human samples, the fluorescence intensities in the acquired images were measured as previously described^17^. Briefly, the kidney was outlined and the intensities were measured in each image using the ImageJ software (U.S. National Institutes of Health, Bethesda, Maryland, USA). To compare the fluorescence intensities in the cortex and medulla in renal biopsy specimens, the PAS staining images of the entire specimen were divided into 8 parts. Each part was evaluated by an experienced nephrologist (T.I.) and defined as cortex or medulla according to the PAS findings. The fluorescence intensities in each part were then measured from the corresponding fluorescence images. The fluorescence intensities in the cortex and medulla were comparatively analysed.

### Statistical analysis

Two-way analysis of variance test was performed to assess the differences in fluorescence intensities between cortex and medulla at 0 min and 3 min. If a significant difference was found, Sidak’s multiple comparison test was performed as post hoc analysis. The unpaired t test was used to assess the differences in the fold increase of fluorescent intensities between cortex and medulla. The difference was considered statistically significant when the P-value was less than 0.05. All values are expressed as mean ± standard error of mean (SEM). All statistical analyses were performed in GraphPad Prism (7.0. for Windows, GraphPad Software, San Diego, CA, USA).

## Supplementary information

Supplementary Information.

## Data Availability

The datasets of this study are available from the corresponding author on reasonable request.
