# Supplementary material for: A novel method for assessing the renal biopsy specimens using an activatable fluorescent probe
Source: Sci Rep. 2020 Jul 21;10:12094. doi: 10.1038/s41598-020-69077-w (PMC7374171; doi:10.1038/s41598-020-69077-w)
Supplement: Supplementary file 1 — Supplementary Information. [file 41598_2020_69077_MOESM1_ESM.pdf]

A novel method for assessing the renal biopsy specimens using an activatable fluorescent probe

Takuji Iyama[1,†], Tomoaki Takata[1,†,*], Kentaro Yamada[1], Yukari Mae[1], Sosuke Taniguchi[1], Ayami Ida[1], Masaya Ogawa[1], Marie Yamamoto[1], Shintaro Hamada[1], Satoko Fukuda[1], Tsutomu Kanda[1], Takaaki Sugihara[1], Hajime Isomoto[1] and Yasuteru Urano[2].

[1]Division of Gastroenterology and Nephrology, Tottori University Faculty of Medicine, 36-1, Nishimachi, Yonago, Tottori, 683-8504, Japan
[2]Laboratory of Chemical Biology and Molecular Imaging, Graduate School of Medicine, The University of Tokyo, 7-3-1 Hongo, Bunkyo-ku, Tokyo, 113-0033, Japan

[†]These authors contributed equally to the study.
* Corresponding to: Tomoaki Takata (email: t-takata@tottori-u.ac.jp)

Supplementary Figure S1.

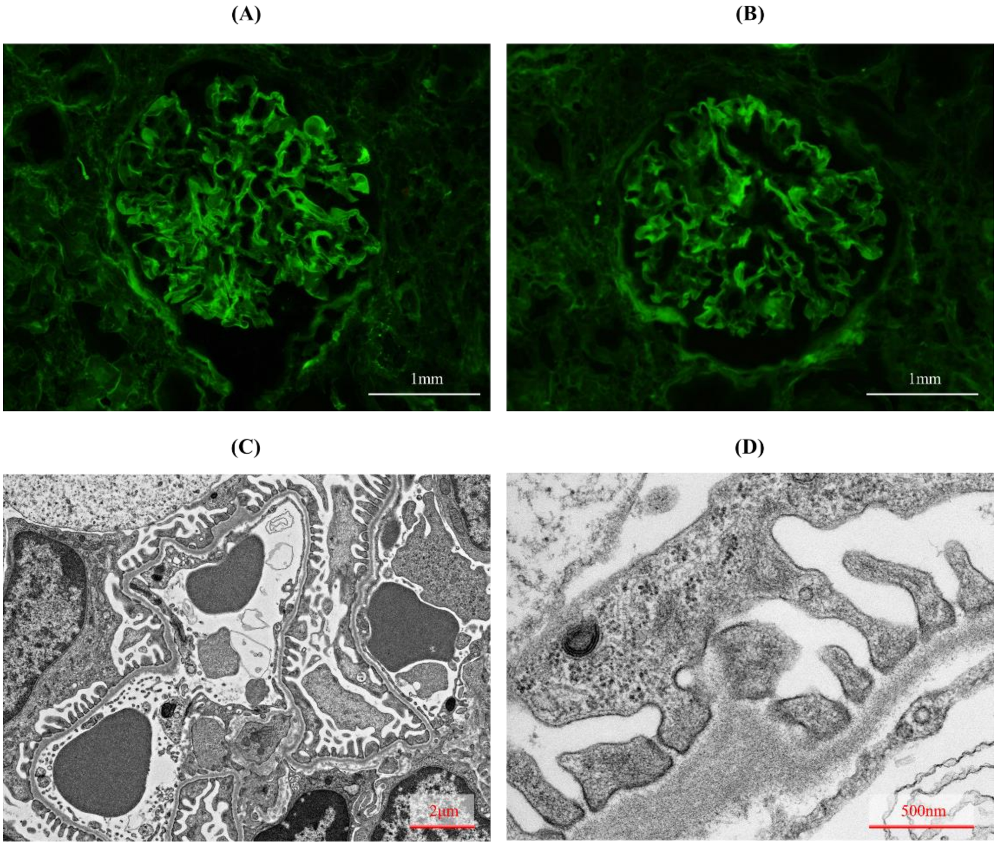

Supplementary Figure S1. Representative immunofluorescence and electron microscope images of human biopsy specimen. Human biopsy specimen was treated with (A) phosphate-buffered saline or (B) gGlu-HMRG for 3min, then 4-μm-thick sections were incubated with FITC-conjugated anti IgG antibody for 30 min at room temperature. Images were obtained using fluorescence microscopy. Linear deposition at the glomerular basement membrane could be observed in both images. (C)(D) Electron microscopy images of mouse kidney treated with gGlu-HMRG for 3min. No obvious structural alteration was observed. FITC; fluorescein isothiocyanate, gGlu-HMRG; γ-glutamyl hydroxymethyl rhodamine green.

Supplementary Figure S2.

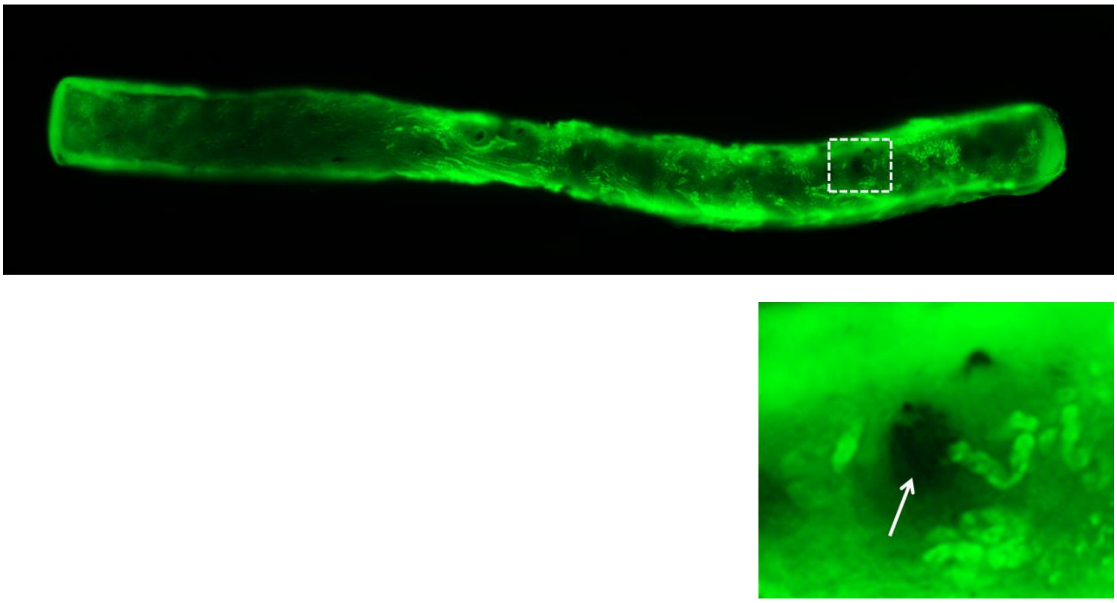

Supplementary Figure S2. Representative fluorescence image of human biopsy specimen. The images were obtained using fluorescence microscope within 3 min post-gGlu-HMRG administration. Glomerulus was devoid of fluorescence signal (arrow). gGlu-HMRG; γ-glutamyl hydroxymethyl rhodamine green.

Supplementary Figure S3.

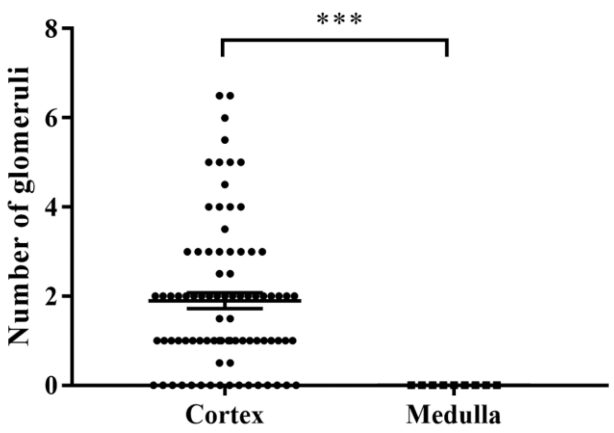

Supplementary Figure S3. Quantification of the number of glomeruli contained in each part. The number of glomeruli contained in each 8 part of the images were counted. Glomerulus was exclusively distributed in the cortex. Data were analysed by unpaired t test. Bars indicate average ± standard error of mean (SEM). ***P<0.001.
